# Determinants of diarrheal diseases among under five children in Jimma Geneti District, Oromia region, Ethiopia, 2020: a case-control study

**DOI:** 10.1186/s12887-021-03022-2

**Published:** 2021-11-30

**Authors:** Dejene Mosisa, Mecha Aboma, Teka Girma, Abera Shibru

**Affiliations:** Department of Public Health, Medicine and Health Sciences College, Ambo University, P.O.BOX:19 Ambo, Oromia Ethiopia

**Keywords:** Unmatched, Case-control, Determinants, Diarrhea, Jimma Geneti, District

## Abstract

**Background:**

Globally, in 2017, there were nearly 1.7 billion cases of childhood diarrheal diseases, and it is the second most important cause of morbidity and mortality among under-five children in low-income countries, including Ethiopia. Sanitary conditions, poor housing, an unsanitary environment, insufficient safe water supply, cohabitation with domestic animals that may carry human pathogens, and a lack of food storage facilities, in combination with socioeconomic and behavioral factors, are common causes of diarrhea disease and have had a significant impact on diarrhea incidence in the majority of developing countries.

**Methods:**

A community-based unmatched case-control study was conducted on 407 systematically sampled under-five children of Jimma Geneti District (135 with diarrhea and 272 without diarrhea) from May 01 to 30, 2020. Data was collected using an interview administered questionnaire and observational checklist adapted from the WHO/UNICEF core questionnaire and other related literature. Descriptive, bivariate, and multivariate binary logistic regression analyses were done by using SPSS version 20.0.

**Result:**

Sociodemographic determinants such as being a child of 12–23 months of age (AOR 3.3, 95% CI 1.68–6.46; *P* < 0.05) and mothers’/caregivers’ history of diarrheal diseases (AOR 7.38, 95% CI 3.12–17.44; *P* < 0.05) were significantly associated with diarrheal diseases among under-five children. Environmental and behavioral factors such as lack of a hand-washing facility near a latrine (AOR 5.22, 95% CI 3.94–26.49; *P* < 0.05), a lack of hand-washing practice at critical times (AOR 10.6, 95% CI 3.74–29.81; *P* < 0.05), improper domestic solid waste disposal (AOR 2.68, 95% CI 1.39–5.18; P < 0.05), and not being vaccinated against rotavirus (AOR 2.45, 95% CI 1.25–4.81; P < 0,05) were found important determinants of diarrheal diseases among under-five children.

**Conclusion:**

The unavailability of a hand-washing facility nearby latrine, mothers’/caregivers’ history of the last 2 weeks’ diarrheal diseases, improper latrine utilization, lack of hand-washing practice at critical times, improper solid waste disposal practices, and rotavirus vaccination status were the determinants of diarrheal diseases among under-five children identified in this study. Thus, promoting the provision of continuous and modified health information programs for households on the importance of sanitation, personal hygiene, and vaccination against rotavirus is fundamental to decreasing the burden of diarrheal disease among under-five children.

## Introduction

The World Health Organization (WHO) defines diarrhea as the passage of three or more loose or liquid stools per day due to an abnormally high fluid content of stool or an abnormal increase in daily stool fluidity, frequency, and volume from what is considered normal for an individual and is caused by bacterial, viral, protozoa, and parasitic organisms [1]. In low-income countries, the two most common etiological agents of moderate-to-severe diarrhea are rotavirus and *Escherichia coli* [2]. Diarrhea is more common when there is a lack of adequate sanitation and hygiene, safe water supply for drinking, cooking, and cleaning, improper feeding practices, and a poor housing situation [3].

Globally, in 2017, a large number of deaths and more than 1.7 billion cases of childhood diarrheal disease occur every year. Sub-Saharan African and South Asian countries account for roughly 80% of morbidity and mortality. According to 2018 WHO reports, in each year, diarrhea kills more than 525, 000 under-5 years’ children. Five countries accounted for 50 % of the deaths, one of which was Ethiopia [4, 5]. Despite the global achievement in the reduction of all-cause of diarrheal diseases, particularly mortality, in the past 30 years, worldwide diarrhea remains the second most important cause of death due to infections among children under 5 years of age [6]. Likewise, in developing countries, childhood mortality is almost 10 times higher than in developed nations. In Africa, it is estimated that children below 5 years old experience a minimum of five episodes of diarrhea a year and about 800,000 children succumb to diarrhea annually [7]. Similarly, diarrheal disease is the most important community health problem in Sub-Saharan African countries and is accountable for greater than 50% of childhood illnesses and 50–80% of childhood deaths in the countries [1, 8]. Ethiopia is one of the emerging sub-Saharan-African countries contributing to the tall burden of diarrheal illness and death [9]. In the year of 2016 alone, generally, 1 in every 15 children dies before reaching their fifth birthday. Among these deaths, diarrhea kills almost fifteen thousand under-five children in Ethiopia [10]. In Ethiopia in particular, diarrheal diseases alone accounted for 23% of the causes of child mortality, which is greater than the annual deaths due to malaria, HIV/AIDS and measles all together [11, 12]. These were due to living conditions, high incidence of illness, lack of safe drinking water supply, sanitation and, hygiene, as well as poorer overall health and nutritional status [1]. In spite of all advances in health technology, improved management, and increased use of oral rehydration therapy in the past decades, diarrheal diseases still continue to be a major cause of morbidity and mortality. Moreover, there is no dramatic change in evidence about whether the health extension program has had an effect on the risk factors of childhood diarrhea [13, 14].

According to the Ethiopia Demographic and Health Surveys (EDHS), under-five mortality declined from 166 deaths per 1000 live births in 2000 to 67 deaths per 1000 live births in 2016. This indicates a 60% decrease in under-five mortality over a period of 16 years. However, the under-five mortality rate in the Oromia regional state was 79 per 1000, which is higher than the national mortality rate. According to this survey, there was no significant change in the prevalence of diarrheal disease among under-five children, which has dropped only from 13% in 2011 to 12% in 2016 [10].

**Fig. 1:**
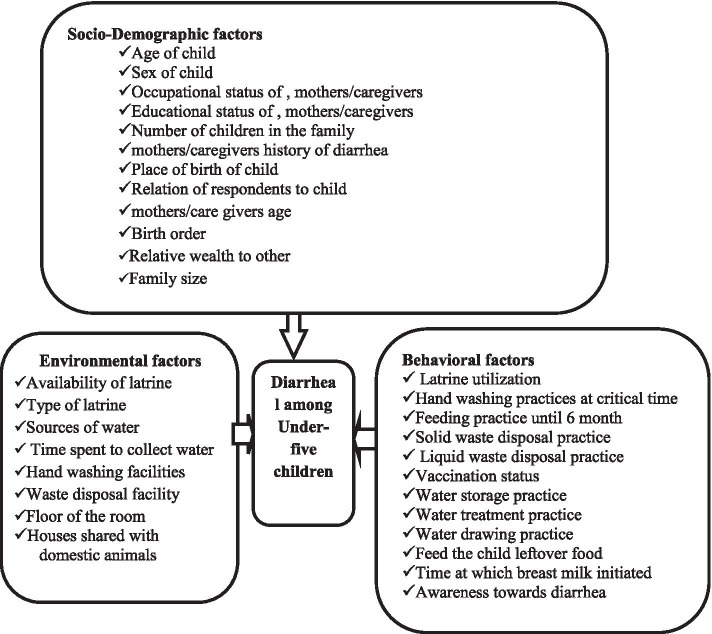
Conceptual framework on Determinants of Diarrheal Diseases among under-five children in Jimma Geneti District, Oromia regional state, Western Ethiopia, May, 2020 [16, 17]

According to the 2019/2020 Jimma Geneti District Health Office performance report, the prevalence of diarrheal diseases among under-five children is 13.5%. Despite the emphasis given by the Ethiopian Federal Ministry of Health, respective regional health offices, Zonal department, and district health offices to improve child health, there is still higher morbidity and mortality among under-five children due to diarrheal disease, specifically in Jimma Geneti District [15]. Generally, the burden of diarrheal diseases in developing countries is associated with different factors. Evidences revealed that, there is a significant variation in the determinants of diarrhea in Ethiopia, i.e., the determinants of diarrhea identified so far by different scholars was not uniform across the districts. Most of the research conducted in Ethiopia was cross-sectional, institutional-based, and used EDHS data to determine its prevalence. While there are insufficient reports on determinants of under-five diarrheal disease in the studied region, and there is no similar study in Jimma Ganti District, where the prevalence of diarrhea is high and higher child mortality and morbidity due to diarrhea were registered. Thus, to achieve the Sustainable Development Goal (SDG) targeting childhood mortality reduction, operational research designed to identify determinants of diarrhea across different geographical settings is required. Therefore, in this study, we aimed to identify determinants of diarrheal disease among under-five children in the Jimma Geneti District, Oromia Region, Ethiopia, which has important public health implications for planning suitable interventions and appropriate strategies to decrease the impact of diarrheal disease [Fig. 1].

## Methods

### Study area and period

**Fig. 2:**
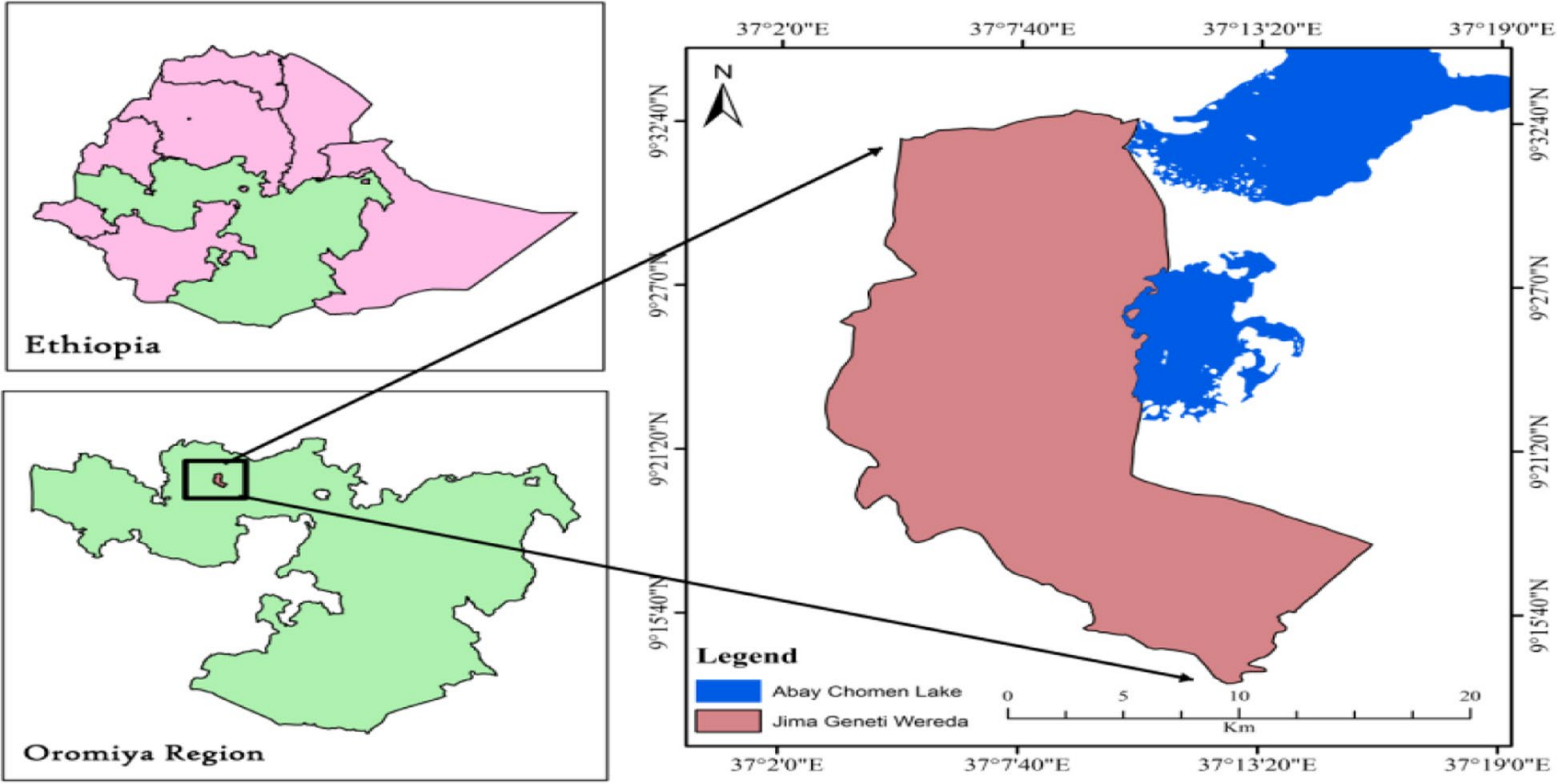
Location map of Jimma Geneti District: Nation, Region and, District, Oromia Regional state, Western Ethiopia, May, 2020 [15]. Source: - Ethiopian Map Agency 2007 [Using GIS Arc map 10.3.1 version 15]

The study was conducted in Jimma Geneti District, from May 01 to 30, 2020. Jimma Geneti District is located in Horo Guduru Wollega Zone, Oromia Regional state, the western part of Ethiopia, 273 km from the Capital City, Addis Ababa. In the district there were 44,278 males and 46,086 females, among whom 5755 (6.4%) were urban and 84,609 (93.6%) were rural, and 18,826 total households. There are 19,998 women of reproductive age and 14,848 under-five children [15] [Fig. 2].

### Study design sample size and sampling procedures

A community-based unmatched case-control study design was conducted to assess determinants of diarrheal diseases among under-five children. The district had 14 kebeles (the district’s smallest administrative unit), four of which were chosen by lottery. The households who had under-five children and residents of the study area in randomly selected kebeles were a sampling unit of this study. While randomly selected under-five children in the households in the preceding 2 weeks before the survey, with a report of diarrhea disease, for cases and without a report of diarrhea disease for controls, were the study units included in this study.

The sample size was determined using OpenEpi’s unmatched case-control model with the assumptions of power = 80%; confidence level = 95%; case to control ratio = 1:2; P1 = proportion of diarrheic children who had not used latrine for disposal of child feces, P2 = proportion of non-diarrheic children who had not used latrine for disposal of child feces as the main predictors of the outcome, which was 33.0 and 19.1% among cases and controls respectively [13]. And an adjusted odds’ ratio (AOR = 2.09) and 10% of none response rates were considered. Finally, a 407 (135 for cases and 272 for controls) sample size was generated.

**Fig. 3:**
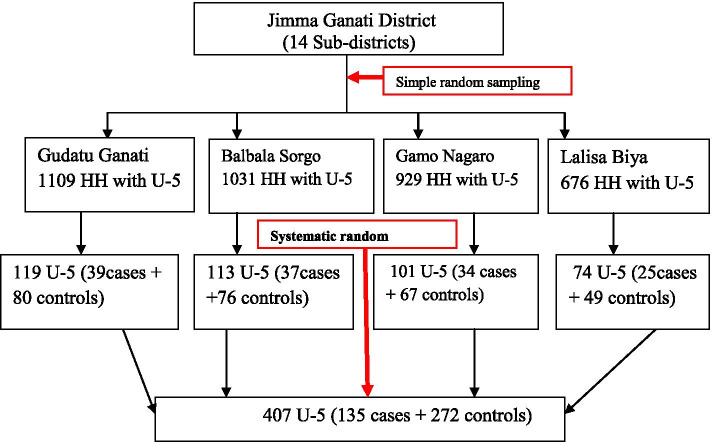
Diagrammatic presentation of sampling technique of under-five children in Jimma Geneti district, Oromia Regional state, Western Ethiopia, May, 2020

A total of 3745 households with under-five children in the selected kebeles were obtained from the family folders of health extension workers (HEWs). Cases and controls were identified by the census through a house-to-house survey, and then 156 under-five children with diarrhea and 3589 under-five children without diarrhea in the selected kebeles were registered and coded with the guidance of HEWs. The case was confirmed from reports of mother’s/caregiver’s history of last 2 weeks period of diarrhea. Afterward, the calculated sample size for control was proportionally allocated to the size of household with under-five children for each selected kebeles. Finally, a total of 272 controls were selected by using the systematic random sampling technique, and all the registered 135 cases who fulfilled the inclusion criteria were included in the study [Fig. 3]. Both cases and controls were recruited from different households, and when there were more than one under-five child in the same household, the youngest child was included in the study since they are more vulnerable to the outcome variable [1].

### Data collection tool and personnel

Data was collected by eight trained B.Sc. nurses under the supervision of four health officers and with guidance of HEWs using a pretested structured questionnaire adapted from the WHO/UNICEF core questionnaire and other related literature [16–18]. In addition, an observational checklist was used to observe water storage containers, the presence or absence of feces around the latrine and compound, availability, and types of the latrine, and the presence or absence of hand-washing facilities nearby the latrine.

### Data quality control and analysis

Data quality was assured through pre-test on 5% of the total sample size in different sub-districts of the study area. Data collectors and supervisors were trained for 1 day by the principal investigator on the study instruments and consent form, how to interview and data collection procedures. The data collection processes were closely supervised by supervisors and investigators. Before data entry, the questionnaires were checked for completeness, consistency, and correction measures made by supervisors and investigators. Then, the data was coded and entered into Epi Info and was exported to SPSS for data processing, cleaning, and analysis. Descriptive analysis like frequency and percentage was carried out to describe sociodemographic characteristics of the respondents and environmental and behavioral determinants of diarrhea among under-five children and results were presented in texts and tables. Bivariate and multivariate analyses were done using a binary logistic regression model to identify determinants of diarrheal diseases among under-five children. Candidate variables for the final model (multivariate logistic regression) were identified using a bivariate logistic regression model with *P* < 0.25. Multivariate logistic regression was used to determine the independent effect of each explanatory variable on the study variable, with significance set to *P* < 0.05.

The Hosmer and Lemeshow goodness-of-fit (*P*-value = 0.348) was checked to test for model fitness. The independent variables were tested for multi-co-linearity using the Variance Inflation Factor (VIF) and the Tolerance tests, and no variables were found to have a VIF greater than 2 to be omitted from the analysis.

### Terms and operational definition

#### Diarrhea

Is defined as having three or more loose or watery stools in a 24-h period in the household within the two-week period before the survey is administered, as reported by the mothers/caregivers of the child [10].

#### Mothers/caregivers

Parried-child mother/caregivers, a person who is responsible for taking care of a child; the person can be a relative of the child or a non-relative.

#### Relative wealth to others

Households are categorized based on the number and kinds of domestic animals they own, ranging from a hen to a cow or ox, in addition to farmland ownership, with the amount of productivity per year and housing characteristics such as consumer goods, toilet facilities, and flooring materials. Each household was ranked by their living standard, and then the distribution was divided into three categories: model, middle, and poor [19].

#### Improved water sources

It includes piped water into the dwelling, piped water to the yard, tube well, or borehole, public standpipes, protected dug wells, protected springs, and rainwater. An improved source is one that is likely to provide “safe” water [6].

#### Improper waste disposal

Is the disposal of waste in a way that has an impact on the environment. Examples include littering, hazardous waste that is dumped into the ground, and not recycling and disposing of refuse in open fields [6].

#### Hand-washing during the critical time

Refers to mothers’/caregivers’ hand-washing practice after the utilization of the latrine, after helping their child defecate, before food preparation, and before self-feeding and child-feeding. If yes, for all critical times of hand-washing, it is concluded as good, otherwise poor practice.

#### Proper latrine utilization

Households with functional latrines and at least no observable feces in the compound, observable fresh feces through the squat hole, and the footpath to the latrine were uncovered with grasses.

#### Good awareness towards diarrhea

mothers/caregivers who mentioned at least three causes of diarrhea such as microorganisms, flies, contaminated food/water, three ways of transmission such as by eating contaminated food, by flies, and by physical contact with the diseased person and its prevention such as vaccination of rotavirus vaccine, early initiation, and exclusive breastfeeding, use safe water for drinking and food preparation, proper waste disposal.

## Results

### Sociodemographic characteristics of study participants

Totally, 407 under five-children (135 cases and 272 controls) were sampled for this study. However, data gathered from 399 under-five children of study participants (127 among cases and 272 among controls) showed a response rate of 94% for cases and 100% for controls. Among those studied children, 76 (59.8%) of cases and 156 (57.4%) of controls were male children and 44 (34.6%) cases and 128 (47.1%) controls were found in the age group of 24–59 months. The mean (+SD) of the age of cases and controls was 18.79 (+ 5.2) and 21.09 (+ 5.9) months respectively. Among these children, 107 (84.3%) of cases and 231 (84.9%) of controls were born at the health facility.

Of all the mothers/caregivers, 118 (92.9%) among cases and 266 (97.8%) among controls were biological mothers. Out of the total mothers/caregivers, 106 (81.9%) cases and 247 (87.9%) controls were found in the age group of 25–35 years.

The majority of mothers/caregivers, 108 (85%) cases, and 201 (73.9%) controls were housewives by occupation. Most of the mothers/caregivers, 115 (90.6%) cases, and 255 (93.8%) controls were married. More than half of the mothers/caregivers in both study groups; 69 (54.3%) cases and 151 (55.5%) controls, had no formal education.

Out of the total, 90 (70.9%) of the mothers/caregivers of the cases and 196 (72.1%) of the controls were protestant religious followers. By ethnicity, approximately 126 (99.2%) of cases and 267 (98.2%) of controls were Oromo.

Regarding the family size of the households in both groups, 62 (48.8%) of cases and 139 (51.1%) of controls were had > = 5 members and the number of under-five children in the households in both groups was one among more than half of the households, 65 (51.2%) of cases and 153 (56.2%) of controls.

Among all households, 34 (26.8%) mothers/caregivers of cases and 14 (5.1%) mothers/caregivers of controls had last two-week history of diarrhea (Table 1).

**Table 1:** Socio-demographic characteristics of study participants in Jimma Geneti District, Oromia Regional state, Western Ethiopia, May, 2020

Socio-demographic characteristics of study participants (*n* = 399)	Frequency	
	Number/Percent of cases (*n* = 127)	Number/Percentage of controls (*n* = 272)
**Sex of Child**		
Male	76 (59.8)	156 (57.4)
Female	51 (40.2)	116 (42.6)
**Age of child**		
0–5 months	12 (9.4)	17 (6.3)
6-11 months	27 (21.3)	48 (17.6)
12–23 months	44 (34.6)	79 (29)
24–59 months	44 (34.6)	128 (47.1)
**Place of Delivery**		
Health facility	107 (84.3)	231 (84.9)
Home	20 (15.3)	41 (15.1)
**Age of the respondents**		
18–24 years	2 (1.6)	9 (3.3)
25–35 years	104 (81.9)	239 (87.9)
> 35 years	21 (16.5)	24 (8.8)
**Relation of the respondents**		
Mother	118 (92.9)	266 (97.8)
Caregiver	9 (7.1)	6 (2.2)
**Ethnicity of respondents**		
Oromo	126 (99.2)	267 (98.2)
Amhara/other	1 (0.8)	5 (1.8)
**Marital status**		
Married	115 (90.6)	255 (93.8)
Single	10 (7.9)	11 (4)
Divorced/Widowed	2 (1.6)	6 (2.2)
**Education status**		
No formal Education	69 (54.3)	151 (55.5)
Grade 1–8	39 (30.7)	63 (23.2)
Grade 9–12	13 (10.2)	34 (12.5)
Grade12+	6 (4.7)	24 (8.8)
**Occupational status**		
Housewife	108 (85.0)	201 (73.9)
Government employee	3 (2.4)	18 (6.6)
Private/other	16 (12.6)	53 (19.5)
**No of U-5 children in HH**		
1	65 (51.2)	153 (56.2)
> =2	62 (48.8)	119 (43.8)
**Relative wealth to other**		
Poor	25 (19.7)	59 (21.7)
Middle	65 (51.2)	140 (51.5)
Model	37 (29.1)	73 (26.8)
**Mothers/caregivers history of diarrhea**		
Yes	34 (26.8)	14 (5.1)
No	93 (73.2)	258 (94.9)

### Environmental related characteristics of study participants respondents

The majority of households, 117 (92.1%) among cases and 258 (94.9%) among controls, had latrine facilities in their compound. From these households that had latrines, more than half, 66 (56.4%) among cases and 160 (62.0%) among controls, used pit latrines without a slab.

About 92 (72.4%) of cases and 201 (73.9%) of controls of households were used improved sources of water supply, and 36 (28.3%) of cases and 87 (32.0%) of controls of households traveled more than 30 min to collect water from the sources.

More than half of households with latrines, 73 (57.5%) of cases and 163 (59.9%) of controls, had a hand-washing facility, and 70 (55.1%) of cases and 163 (59.9%) of controls had a waste disposal facility in their compound.

The majority of the floors of the households, 94 (74.0%) of cases, and 214 (78.7%) of controls, were made of soil. About 112 (88.2%) of cases and 258 (84.9%) of controls of households had separated kitchens from their houses. From the total households, 104 (81.9%) of the cases and 251 (92.3%) of the controls were not shared houses with domestic animals (Table 2).

**Table 2:** Environmental related characteristics of study participants in Jimma Geneti District, Oromia Regional State, Western Ethiopia, May, 2020 (*n* = 399)

Environmental related characteristics of study participants (*n* = 399)	Frequency	
	Number/percent of cases (*n* = 127)	Number/percentage of controls (*n* = 272)
**Latrine availability**		
Yes	117 (92.1)	258 (94.9)
No	10 (7.9)	14 (5.1)
**Types of latrine**		
Pit latrine without slab	66 (56.4)	160 (62.0)
Pit latrine with slab	7 (6.0)	41 (16.0)
Ventilated improved pit latrine	44 (37.6)	57 (22.0)
**Sources of water**		
Improved	92 (72.4)	201 (73.9)
Unimproved	35 (27.6)	71 (26.1)
**Time spent to collect water**		
</= 30 min	91 (71.7)	185 (68.0)
> 30 min	36 (28.3)	87 32.0)
**Availability of hand-washing facility**		
Yes	73 (57.5)	173 (63.6)
No	54 (42.5)	99 (36.4)
**Availability of waste disposal facility**		
Yes	70 (55.1)	163 (59.9)
No	57 (44.9)	109 (40.1)
**Ownership status of the house**		
Private	103 (81.1)	219 (80.5)
Rented	24 (18.9)	53 (19.5)
**Floor of house**		
Soil	94 (74.0)	214 (78.7)
Wood	3 (2.4)	17 (6.3)
Cement	30 (23.6)	41 (15.1)
**Availability of separated kitchen**		
Yes	112 (88.2)	258 (84.9)
No	15 (11.8)	14 (5.1)
**Houses shared with domestic animals**		
Yes	23 (18.1)	21 (7.7)
No	104 (81.9)	251 (92.3)

### Behavioral characteristics of study participants

Regarding behavioral characteristics’ majority of households, 75 (59.1%) among cases, and 220 (80.9%) among controls were properly practiced latrine utilization. Greater than three fourth, 102 (80.3%) among cases and 265 (97.4%) among controls of respondents have washed their hands at critical times. Sixty-four (50.4%) of households from cases and 176 (64.7%) of households from controls were disposed domestic solid refuse properly, while 65 (51.2%) from cases and 113 (41.5%) from controls were disposed of liquid waste improperly.

More than half of under-five children, 73 (62.9%) from cases and 198 (77.6%) from controls, were vaccinated for the measles vaccine. And 73 (57.5%) of cases and 208 (76.5%) of controls were received rotavirus vaccine. From all mother/caregivers, 78 (61.4%) among cases and 171 (62.9%) among controls had good awareness towards diarrheal morbidity (Table 3).

**Table 3:** Behavioral characteristics of study participants in Jimma Geneti District, Oromia Regional State, Western Ethiopia, May, 2020 (*n* = 399)

Behavioral characteristics of study participants (*n* = 399)	Frequency	
	Number/Percentage of cases (*n* = 127)	Number/Percentage of controls (*n* = 272)
**Latrine Utilization**		
Proper utilization	75 (59.1)	220 (80.9)
Improper utilization	52 (40.9)	52 (19.1)
**Hand washing at critical time**		
Yes	102 (80.3)	265 (97.4)
No	25 (19.7)	7 (2.6)
**Feeding practice until 6 months**		
Exclusive breastfeeding	99 (78.0)	245 (90.1)
Mixed feeding	26 (20.5)	24 (8.8)
Formula feeding	2 (1.6)	3 (1.1)
**Feed the child leftover food**		
Yes	13 (10.2)	15 (5.5)
No	114 (89.8)	257 (94.5)
**Solid waste disposal**		
Proper	64 (50.4)	176 (64.7)
Improper	63 (49.6)	96 (35.3)
**Liquid waste refusal**		
Proper	62 (48.8)	159 (58.5)
Improper	65 (51.2)	113 (41.5)
**Measles Vaccine**		
Vaccinated	73 (62.9)	198 (77.6)
Unvaccinated	43 (37.1)	57 (22.4)
**Rotavirus Vaccine**		
Vaccinated	73 (57.5)	208 (76.5)
Unvaccinated	54 (42.5)	64 (23.5)
**Water treatment at home**		
Yes	49 (38.6)	154 (56.6)
No	78 (61.4)	118 (43.4)
**Ways of collected water drawn**		
By dipping	22 (17.3)	33 (12.1)
By pouring	105 (82.7)	239 (87.9)
**Time of initiating breastfeeding**		
Within one hour	103 (81.1)	231 (84.9)
After one hour	24 (18.9)	41 (15.1)
**Awareness towards diarrhea**		
Good	78 (61.4)	171 (62.9)
Poor	49 (38.6)	101 (37.1)

### Determinants of diarrheal disease among under-five children

The result of backward likelihood multivariate logistic regression analysis revealed that age of child, availability of hand-washing facility, nearby latrine, mothers’/caregivers’ history of the last 2 weeks’ diarrheal disease, latrine utilization, hand-washing practice during a critical time, domestic solid waste refusal practice, and rotavirus vaccination status showed that they were statistically significantly associated with diarrheal diseases among under-five children, after controlling for potential confounders.

Thus, the odds of developing diarrheal disease among under-five children were 2.5 and 3 times higher among children of age 6–11 and 12–23 months, respectively, as compared to children of age 24–59 months (AOR 2.46; 95%CI: 1.09–5.57 and AOR 3.3; 95%CI: 1.68–6.46; P 0.05).

When compared to counterparts, the odds of developing diarrheal disease among under-five children from households with no hand-washing facility near their latrine were five times higher (AOR 5.2; 95% CI: 3.94–26.49; P 0.05). Under-five children whose mothers’/caregivers’ had a history of diarrheal disease in the last 2 weeks had 7 times more likely to develop the disease as compared with their counterparts (AOR 7.38; 95%CI: 3.12–17.44; *P* < 0.05).

The odds of developing diarrheal disease among under-five children was about 2 times higher among households who had not utilized latrines properly when compared to households who had properly utilized them (AOR 2.34; 95%CI: 1.16, 4.75; *P* < 0.05). The odds of developing diarrheal disease were 10.6 times higher among under-five children whose mothers’/caregivers’ did not wash their hands during critical time compared with under-five children whose mothers’/caregivers’ did wash their hands during critical times (AOR 10.6; 95%CI: 3.7–29.8; *P* < 0.05).

Odds of developing diarrheal disease among under-five children whose mothers/caregivers practiced improper domestic solid waste disposal were about 2.7 times higher than under-five children whose mothers’/caregivers’ practiced proper domestic solid waste disposal (AOR 2.68; 95%CI: 1.39–5.18; *P* < 0.05).

Unvaccinated under-five children were 2.5 times more likely to develop diarrhea disease compared to rotavirus vaccinated children, (AOR 2.45; 95%CI: 1.25–4.81 mothers’/caregivers’) (Table 4).

**Table 4:** Determinants of diarrheal disease among under-five children in Jimma Geneti District, Oromia Regional State, Western Ethiopia, May 2020 (*n* = 399)

Variables	Diarrheal diseases status among under-five children				
	Case = 127, No (%)	Control = 272, No (%)	COR (95%CI)	AOR (95%CI)	*P*-Value
**Age of child**					
0–5 months	12 (9.4)	17 (6.3)	1.88 (0.82, 4.33)+	1.48 (0.61,3.61)	.387
6-11 months	27 (21.3)	48 (17.6)	1.64 (0.91, 2.93)+	2.46 (1.09,5.57)*	.030
12–23 months	44 (34.6)	79 (29)	1.66 (1.00, 2.74)*	3.30 (1.68,6.46)*	.001
24–59 months	44 (34.6)	128 (47.1)	1.00		
**Relation**					
Mother	118 (92.9)	266 (97.8)	1.00	1.00	
Caregiver	9 (7.1)	6 (2.2)	3.38 (1.18, 9.72)*	0.58 (0.32,1.06)	.073
**Hand washing facility**					
Yes	73 (57.5)	173 (63.6)	1.00	1.00	
No	54 (42.5)	99 (36.4)	1.29 (0.84, 1.99)+	5.2 (3.94,26.49)**	<.001
**Mother/care givers’ history of diarrhea**					
Yes	34 (26.8)	14 (5.1)	6.74 (3.46, 13.1)*	7.38 (3.1,17.44)**	<.001
No	93 (73.2)	258 (94.9)	1.00	1.00	
**Latrine Utilization**					
Proper	75 (59.1)	220 (80.9)	1.00	1.00	
Improper	52 (40.9)	52 (19.1)	2.93 (1.84, 4.67)*	2.34 (1.16,4.75)*	.018
**Critical time Hand washing**					
Yes	102 (80.3)	265 (97.4)	1.00	1.00	
No	25 (19.7)	7 (2.6)	9.28 (3.89, 22.1)*	10.6 (3.7,29.8)**	<.001
**Solid waste disposal**					
Proper	64 (50.4)	176 (64.7)	1.00	1.00	
Improper	63 (49.6)	96 (35.3)	1.81 (1.18, 2.77)*	2.68 (1.39,5.18)*	.003
**Rotavirus Vaccine**					
Vaccinated	73 (57.5)	208 (76.5)	1.00	1.00	
Unvaccinated	54 (42.5)	64 (23.5)	2.40 (1.53,3.77)*	2.45 (1.25,4.81)*	.009
**Water treatment**					
Yes	49 (38.6)	154 (56.6)	1.00	1.00	
No	78 (61.4)	118 (43.4)	2.08 (1.35, 3.19)*	1.06 (0.57,1.97)	.867

Case = under-five children with diarrhea, Control = under-five children without diarrhea, Crude odds’ ratio (COR), adjusted odds’ ratio (AOR), Confidence interval (CI), *P*-value derived from multivariate logistic regression based on likelihood ratio test, significant CI of the models are indicated in the bold letter, *p < 0.05; **p < 0.001.

## Discussion

The result of this study showed that children’s age groups 6–11 and 12–23 months were 2.5 and 3 times more likely to develop diarrhoea disease as compared to children in the age group 24–59 months, respectively. This result was consistent with the results of other case-control studies conducted in Medebay Zane District, Gobi District, and Rural Ethiopia [20–22].

Similarly, this result was consistent with the study reported from Indonesia and Guatemala [23, 24]. In general, children older than 24 months had a lower risk of having diarrheal diseases than children whose ages were between 6 and 23 months. The likely explanation for this risk might be that children between the ages of 6–23 months are undergoing complementary feeding, which may make them vulnerable to diarrheal disease-causing infectious agents due to their undeveloped immunity. Moreover, children at these ages are starting to crawl and walk, thus they may pick dirty or other contaminated objects and take them to their mouth. Likewise, the 2016 EDHS report revealed that diarrhoea prevalence remains high (18%) at the age of 12–23 months, for the reason that weaning and walking often occur during these ages, which contribute to the increased risk of contamination from the environment [10].

The unavailability of a hand-washing facility near the latrine was positively associated with childhood diarrheal disease. In this study, under five-year-old children from households that had no hand-washing facilities adjacent to the latrines were about five times more likely to have diarrheal diseases than under-five-year-old children from households that had hand-washing facilities adjacent to the latrines. The result of this study was consistent with the study conducted in Jimma District and Yama Gulale [25, 26]. This might be expressed as where the hand-washing facilities were unavailable near the toilet; the mothers/caregivers may not frequently practice hand-washing after using the toilet and unintentionally feed their children with contaminated hands, which could be contributing to the high prevalence of under-five diarrheal diseases in the district.

Additionally, the findings of this study showed that mothers’/caregivers’ history of diarrheal diseases was significantly associated with diarrhea diseases among under-five children. Children whose mothers/caregivers had diarrheal diseases in the last 2 weeks prior to this study were 7 times more likely to develop diarrheal diseases than children whose mothers/caregivers had no history of diarrheal diseases in the last 2 weeks. The result of this study was similar to the study findings conducted in Ethiopia Harar Town, Medebay Zana District, and Pawi Hospital, Northwest Ethiopia [14, 20, 27]. The fact is that mothers/caregivers are the main food handlers in the family and the main childcare providers; hence, the possibility of diarrheal diseases among children with mothers/caregivers who have had diarrheal diseases is a common event. It also indicates poor hygienic practice in the household results in the occurrence of diarrheal diseases among under-five children. This might be due to mothers/caregivers with diarrheal diseases being considered as a source of infection for diarrheal diseases among under-five children. Moreover, the mother/caregivers might not be providing appropriate and comprehensive care for the child, which could be a contributing factor to the overall burden of under-five diarrhea and its consequences in the study area. The result of this study also revealed that households who improperly utilized latrines were 2 times more likely to develop diarrheal diseases among under-five children compared to households that utilized latrines properly. The result of this study was comparable with the study findings reported from West Gojjam, Ethiopia [13] and the Kawangware Slum in Nairobi County, Kenya [28]. This showed that the presence of a latrine alone does not ensure the prevention of diarrheal diseases among under-five children unless properly utilized. Many microorganisms that cause diarrheal diseases may be controlled when latrines are used properly.

This study found that children whose mothers/caregivers did not practice hand-washing during the critical period were 10.6 times more likely to be affected by diarrheal disease than children whose mothers/caregivers did practice hand-washing during the critical times. This finding was in line with the studies conducted in Adama Rural and Harena Buluk woreda in Ethiopia [29, 30] and in Zambia [31]. This might indicate that diarrheal diseases are largely spread through contaminated hands, water and food supplies. This contamination occurs mainly from inadequate hygiene and sanitation. Contaminated hand is the main source of infection thus; mothers/caregivers should wash their hands at a critical time to prevent diarrheal diseases.

The findings of this study revealed that improper domestic solid waste disposal practices were 2.7 times more likely to be at risk of developing diarrhea diseases compared to their counterparts. The results of this study were consistent with the studies conducted in the Medebay Zana District and Jamma District in Ethiopia [20, 26] and in Kenya [28]. This might be due to improper disposal of domestic solid waste, which serves as a source of infectious agents and reproduction sites for insects. As well, improper domestic solid waste disposal practices create a favorable environment for flies that carry pathogens and could be sources of contamination for water, food, and food utensils. These might cause children to be exposed to contaminated environments and are a leading risk factor for diarrheal diseases among under-five children.

The result of our study finding indicated that children who were not received the rotavirus vaccine were 2.5 times more likely to develop diarrheal diseases as compared to those children who were received the rotavirus vaccine. This finding was in line with the studies conducted at Harena Buluk Woreda, Bahir Dar, and Debre Berhan in Ethiopia [29, 32, 33] and in sub-Saharan Africa countries, Cameroon, and Madagascar [34, 35]. These findings were reported that the rotavirus vaccine showed a significant association with the occurrence of diarrheal diseases among under-five children. This confirmed that the rotavirus vaccination is one of the best ways to prevent diarrheal morbidity and its consequences, together with improvements of sanitation and hygienic practices. Thus, two-dose rotavirus vaccines should be given for children as part of a comprehensive approach to control diarrhea. Evidence from experts review on vaccines suggests that rotavirus vaccines effectiveness provide sufficient prevention against rotavirus episodes among under-five children thus reducing the morbidity of diarrhea among this age group [36, 37].

### Strength and limitation of the study

One of the strengths of this study was that it was conducted community-based using a case-control study design and using the WHO/UNICEF core-based standard questionnaire for data collection. Some behavioral practices, including hand-washing practices at a critical time, reports of mother’s/caregiver’s history of the last 2 weeks of diarrhea, and treatment of drinking water at home used in the analysis were self-reported by the mothers/caregivers, which might introduce imprecision and information bias. Not including data on breastfeeding status, HIV sero-status and social factors could be considered as an additional limitation of this study.

## Conclusion

The unavailability of a hand-washing facility nearby latrine, mother’s/caregiver’s history of the last 2 weeks’ diarrheal diseases, improper latrine utilization, lack of hand-washing practice at a critical time, improper solid waste disposal practices, and rotavirus vaccination status were the determinants of diarrheal diseases among under-five children identified in this study. Most of the identified determinants of diarrheal disease among under-five children in the study area are preventable. Thus, promoting the provision of continuous and modified health information programs for households on the importance of sanitation (proper domestic solid waste disposal and latrine utilization), personal hygiene (hand-washing facilities and proper hand-washing practices at critical times), and vaccination against rotavirus are fundamental to decreasing the burden of diarrheal disease among under-five children.

### Recommendations

The District Health Office and Zonal Health Department should encourage the community to install a hand-washing facility nearby the latrine, motivate the community to utilize the latrine properly and practice hand-washing during a critical time, and strengthen rotavirus vaccination for all under-five children.

Health Extension Workers should facilitate and give health information to mothers/caregivers on the importance of the availability of hand-washing facilities near the latrine, personal hygiene, and proper latrine utilization, hand-washing practices during a critical time, proper solid waste disposal practices, vaccination of rotavirus, and homemade drinking water treatment practices. Local NGOs should collaborate with the District Health Office and other stakeholders on the construction of nearby hand-washing facilities, personal hygiene to prevent the transmission of diarrhea disease from mother to child, the introduction of hand-washing practices at a critical time, and the preparation of areas for proper solid waste disposal practices.

## Data Availability

The dataset used and analyzed throughout the present study accessible from the corresponding author based on reasonable request.

## Abbreviations

AIDS: Acquired immunodeficiency syndrome
AOR: Adjusted odds ratio
CI: Confidence interval
COR: Crude odds ratio
EDHS: Ethiopian demographic and health survey
HH: Households
HO: Health officer
PI: Principal investigators
SD: Standard deviation
SDG: Sustainable development goal
SPSS: Statistical package for social science
SSA: Sub Saharan Africa
U-5: Under five years old children
UNICEF: United Nations Children’s Fund
